# The diet and benign paroxysmal positional vertigo (DaBC) study: protocol and baseline characteristics of a prospective cohort investigating dietary patterns and BPPV prognosis—the role of genetics and gut microbiota

**DOI:** 10.3389/fnut.2025.1640153

**Published:** 2025-09-15

**Authors:** Juanli Xing, Hongying Shan, Xinyu Xu, Wenyan Shi, Peihua Ren, Jiaqian Wu, Le Ma, Baibing Mi

**Affiliations:** ^1^Department of Otolaryngology-Head and Neck Surgery, The First Affiliated Hospital of Xi'an Jiaotong University, Xi'an, China; ^2^Department of Epidemiology and Biostatistics, School of Public Health, Xi'an Jiaotong University Health Science Center, Xi'an, Shaanxi, China; ^3^Department of Occupational and Environmental Health, School of Public Health, Health Science Center, Xi'an Jiaotong University, Xi'an, China; ^4^The First Affiliated Hospital of Xi'an Jiaotong University, Xi'an, China; ^5^The Second Affiliated Hospital, Xi'an Jiaotong University, Xi'an, China

**Keywords:** benign paroxysmal positional vertigo, cohort study, dietary patterns, baseline characteristics, study protocol

## Abstract

**Background:**

As the prevalent cause of dizziness, benign paroxysmal positional vertigo (BPPV) is increasingly considered a major public health concern due to its high recurrence rate and persistent symptoms. Growing evidence suggests a biologically plausible link between dietary factors and BPPV progression. However, current research on the role of diet in BPPV has predominantly focused on individual nutrients and disease onset, with limited evidence regarding the impact of overall dietary patterns on post-treatment clinical outcomes, especially in Asian populations. Furthermore, the potential interactions among diet, genetic predispositions, and gut microbiota in relation to BPPV prognosis remain insufficiently understood and warrant further investigation. In this study, we conducted a prospective cohort of patients with BPPV in the Chinese population to evaluate the association between post-treatment dietary patterns and their changes, with the recurrence risk of BPPV, and long-term symptoms after standardized reposition therapy, as well as to investigate the potential modifying roles of genetic variations and gut microbiota.

**Methods/design:**

The Diet and BPPV Cohort Study (DaBC) was initiated in July 2023 and is an ongoing multicenter prospective cohort study conducted at three specialized neuro-otology centers in Northwest China. Participants underwent comprehensive baseline assessments including medical history, otoneurological evaluations, dietary intake via a validated semi-quantitative food frequency questionnaire (FFQ), psychological status, balance function, and biomarker collection (blood and fecal samples for genotyping and gut microbiota profiling). Follow-up assessments will be scheduled at 1 month and annually for 5 years post-baseline. The primary outcome is defined as the number of BPPV relapses during the 5-year follow-up, while secondary outcomes include average recurrence intervals and patient-reported symptom burdens such as dizziness handicap, anxiety, depression, and sleep disturbances. By October 30, 2024, a total of 844 first-diagnosed BPPV patients and complete baseline data were enrolled. We describe the study design and present baseline characteristics of the participants enrolled in the cohort to date.

**Discussion:**

With multi-Omics Framework of DaBC Cohort Study, our future findings are anticipated to yield valuable epidemiological evidence regarding the role of diet in BPPV outcomes, which may provide foundational insights to inform clinical recommendations and refine patient management strategies.

## 1 Introduction

Benign paroxysmal positional vertigo (BPPV) is a dysfunction of the peripheral vestibular system, characterized by brief, recurrent episodes of vertigo and positional nystagmus induced by specific head movements (1). It is estimated that approximately 20%−30% (2) of patients with vestibular symptoms worldwide are affected by BPPV, making it the most prevalent cause of dizziness (3). BPPV significantly impairs daily functioning, increases the risk of falls, and causes psychological distress (4–6); it may also predispose individuals to ischemic stroke, dementia, and severe disability, collectively imposing substantial health and economic burdens (7–10). In the United States, medical costs related to BPPV have reached $2 billion (11) annually and are projected to rise as the population ages. Despite proven benefits in symptom control with treatment targeting BPPV, these interventions are suboptimal in reducing high recurrence rates and improving the quality of life for a considerable proportion of affected individuals (12). After the first-line treatment for BPPV, nearly 30% of patients report recurrence within the 1st year (13, 14) and continue to suffer from persistent symptoms such as residual dizziness, anxiety, and vestibular dysfunction (4, 15, 16). Therefore, identifying potential risk factors associated with recurrence and long-term symptoms of BPPV is critical to delay or prevent the development of the disease.

The pivotal pathological mechanism underlying BPPV recurrence involves the abnormal production and dislodgement of otoconia, which may be associated with increased oxidative stress (17) and microcirculatory disturbances (18). An appropriate diet has been shown to have antioxidant properties and microcirculation-improving effects, suggesting biologically plausible effects of diet on BPPV progression. Prior studies have explored the relationship between dietary factors and BPPV, highlighting the possible key roles of carbohydrates and high-quality fats (19). Existing research predominantly focuses on exploring the dietary habits associated with the onset of BPPV (19); however, little is known about the association of dietary factors and recurrence rates and persistent symptoms, such as balance dysfunction that critically compromises long-term quality of life in BPPV patients (20–22). Although recent meta-analyses have addressed the association between specific nutrients and BPPV recurrence, given the complex interaction of nutrients, isolated nutrients fail to capture the integrated features of diet (4, 19). Healthy dietary patterns, such as the Mediterranean or DASH diets, have been found to drive positive health effects in the prognostic management of chronic diseases (23). However, the association of overall diet with relapse and neurological dysfunction in patients with BPPV has not yet been substantiated. Notoriously, multiple factors, including race, genetic variations, and gut health, may lead to wide-ranging interindividual variability in response to nutrients (24, 25). As such, whether the disparities of genetic variations and gut health are involved in modifying the association of diet and the prognosis in patients with BPPV still deserves further exploration. Furthermore, current evidence mainly originates from studies conducted in Caucasian populations, which may not be directly extrapolated to the Chinese population with distinct allele frequencies in otoconial-related genes (such as OTOG and COL11A2) and unique culturally driven dietary patterns (26, 27).

To address these knowledge gaps, we established a prospective cohort of patients with BPPV in the Chinese population to investigate the association between dietary patterns with the recurrence risk of BPPV, vestibular function recovery, and long-term quality of life after standardized reposition therapy, and to determine whether this association is affected by genetic variations and gut microbiota.

## 2 Materials and methods

### 2.1 Study design and population

**Figure 1:**
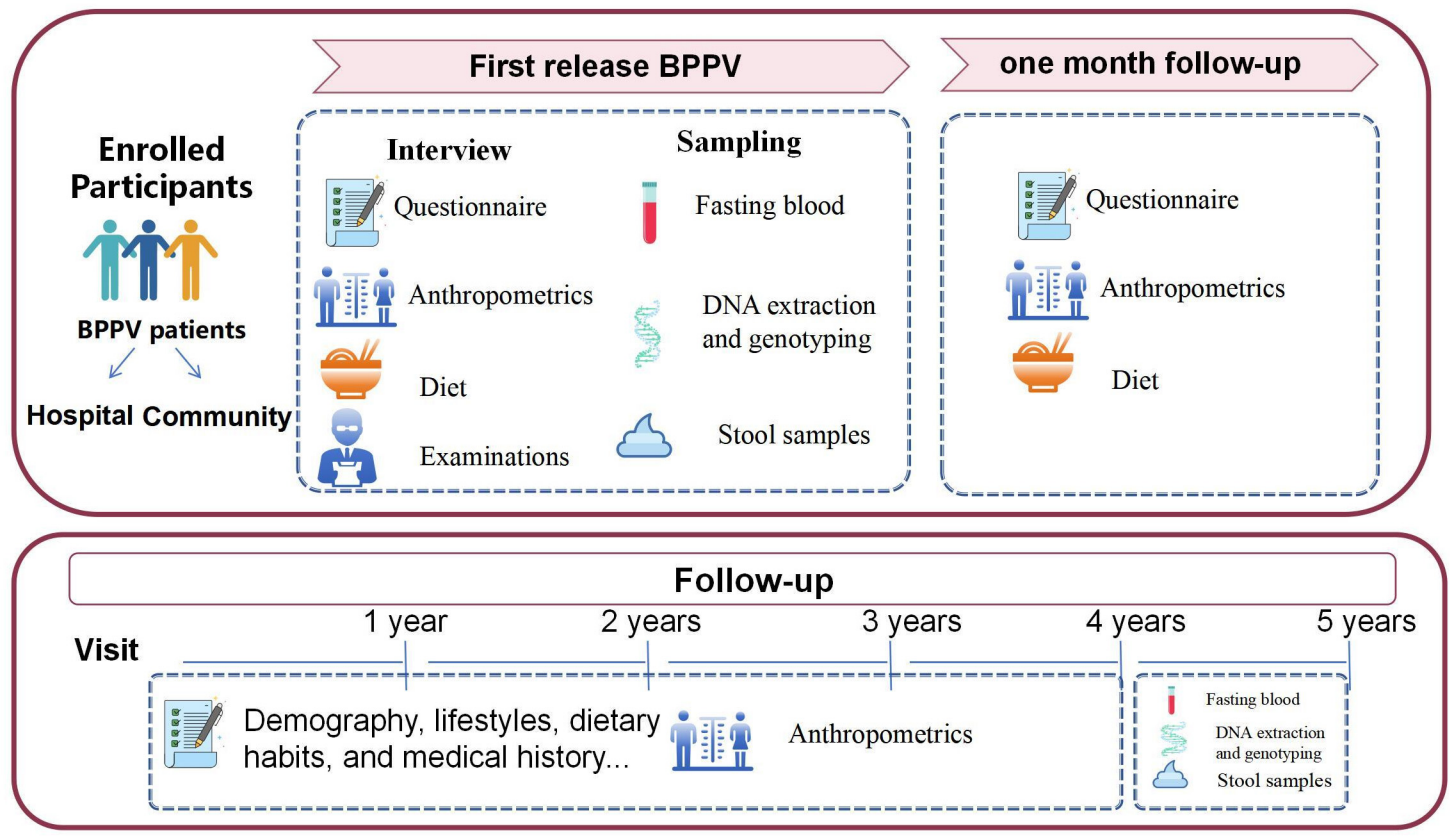
Multi-omics framework of DaBC cohort study.

The Diet and BPPV Cohort Study (DaBC) is an ongoing multicenter prospective cohort study conducted at three specialized neuro-otology centers in Northwest China (Figure 1). Recruitment posters detailing study objectives were displayed in hospital outpatient clinics and community health centers. These posters featured a Quick Response (QR) code linking to a platform for collecting basic demographic information and self-reported dizziness symptoms. Initiated in July 2023, all potential patients involved in the recruitment process were invited to the department of the Otolaryngology-Head (ENT) of the involved centers for a comprehensive clinical evaluation, including an extensive medical history from an otolaryngology head and neck surgeon as well as detailed otologic and neurologic evaluations.

**Figure 2:**
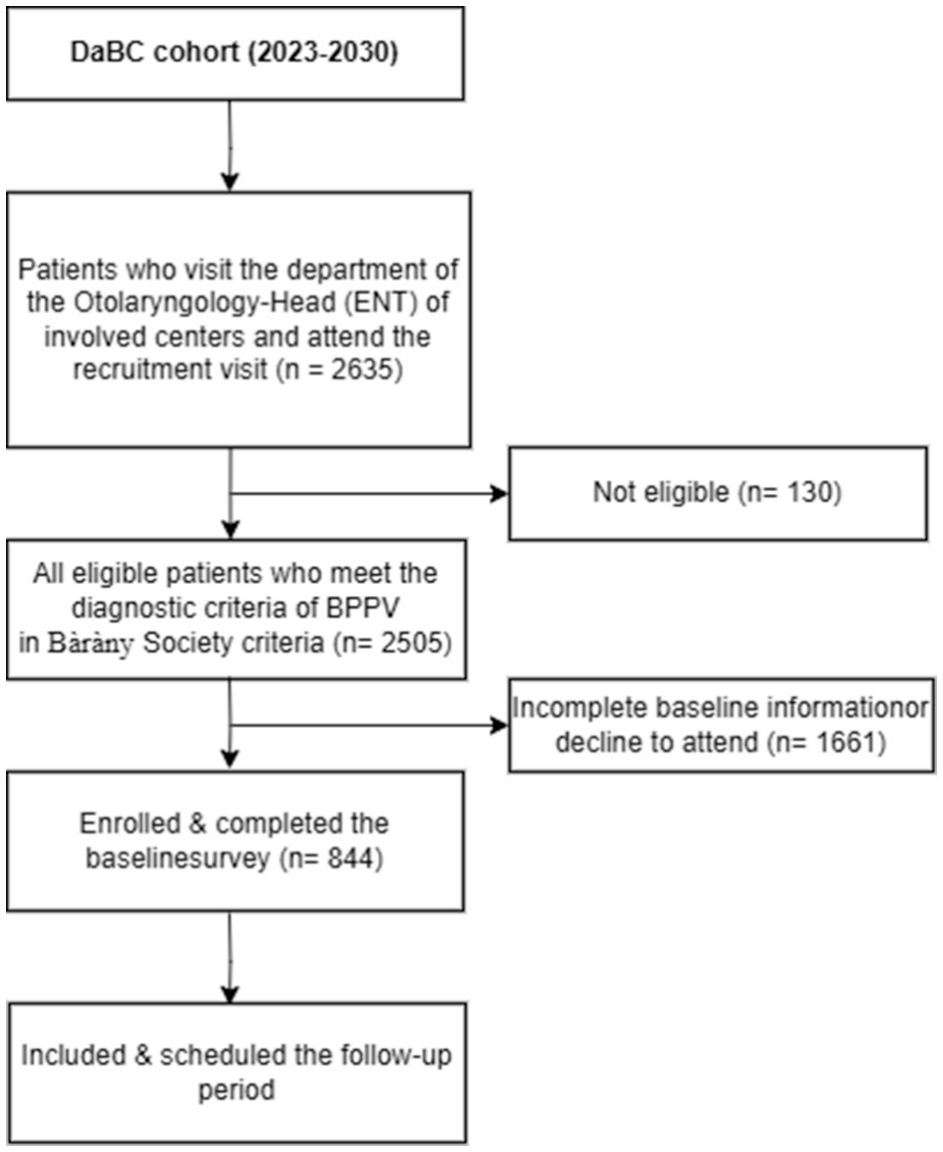
The composition of DaBC cohort 30th March 2025.

Participants are eligible if they are aged 18 years or older and diagnosed with BPPV by an otorhinolaryngologist following the Bàràny Society criteria (2). The diagnosis of BPPV is based on: (1) Presence of episodic positional vertigo of brief duration; (2) Presence of characteristic positioning nystagmus indicative of BPPV, elicited through Dix–Hallpike (posterior canal BPPV) or Supine head roll testing (horizontal canal BPPV) (11). Individuals are excluded if they have physician-diagnosed Meniere's disease, vestibular neuritis, vestibular migraine, or other vestibular diseases; have dizziness attributable to systemic diseases, such as cardiovascular and cerebrovascular disease, endocrine disorders; have a history of head trauma, inner ear surgery, exposure to ototoxic agents or related withdrawal syndromes; have communication impairments. Participants will also be excluded who are unavailable to attend the scheduled follow-up investigation. Initiated in May 2022, the DaBC successfully recruited 2,505 eligible BPPV patients by the closing date of October 30, 2024. Of these, 844 participants with complete baseline data were invited for subsequent follow-up, as outlined in the study protocol (Figure 2).

The study was approved by the Research Ethics Committees of Xi'an Jiaotong University (XJTU2021-1560). Detailed aims and protocol of DaBC will be delineated to all participants, and written informed consent will be obtained at enrollment. The cohort study adheres to the Strengthening the Reporting of Observational Studies in Epidemiology (STROBE) guidelines (28) and the Declaration of Helsinki.

### 2.2 Follow-up procedures

To ensure that the data collected reflects the natural course and initial presentation of BPPV, the participants in this study are restricted to first-diagnosed patients with BPPV. This study plans to enroll 1,200 people with complete baseline information through May 2025. At enrollment, participants are instructed to complete a detailed screening questionnaire, including sociodemographic data, medical history, lifestyle behaviors, anthropometrics, menopausal status and postmenopausal hormone use (for women). Following the screening, thorough history-taking and otolaryngologic, audiological, and neurotological evaluations, especially the Dix–Hallpike maneuver and the Supine head roll test, will be performed to further confirm BPPV diagnosis and determine eligibility of participants (29). Concurrently, participants are also requested to provide dietary information for the past year prior to diagnosis via a validated semi-quantitative Food Frequency Questionnaire (FFQ). Residual dizziness severity of BPPV patients is assessed using the validated 25-item Dizziness Handicap Inventory (DHI). Besides, participants are also asked to complete standardized scales including: the Vestibular Activities of Daily Living Scale (VADL) quantifying functional balance limitations; the Activities-specific Balance Confidence (ABC) Scale measuring self-efficacy in ambulation; the Hospital Anxiety and Depression Scale (HADS) screening psychological comorbidities; and the Pittsburgh Sleep Quality Index (PSQI) evaluating sleep disturbances—collectively measuring multidimensional BPPV-related symptom burdens and quality of life impacts. At the same time, fasting blood samples are collected for genotyping and quantification of circulating nutrients and other potentially bioactive substances; fecal specimens are also collected for gut microbiota profiling.

The follow-up of the DaBC study is performed based on the comprehensive consideration of the follow-up interval of BPPV progression in previous studies. Data were collected at baseline (time of diagnosis), 1 month, and annually through 5 years after the baseline examination (Figure 1). All patients in the cohort study will participate in routine follow-up visits after their initial visit. First, researchers will record the phone numbers and WeChat accounts of the participants for active surveillance of disease progression, dietary adherence, and other health-related information during the 5-year follow-up period. Second, patients will contact their physicians via WeChat upon symptom recurrence to initiate prompt clinical evaluations. Diagnostic tests and symptom assessments will be documented during each unscheduled visit.

**Figure 3:**
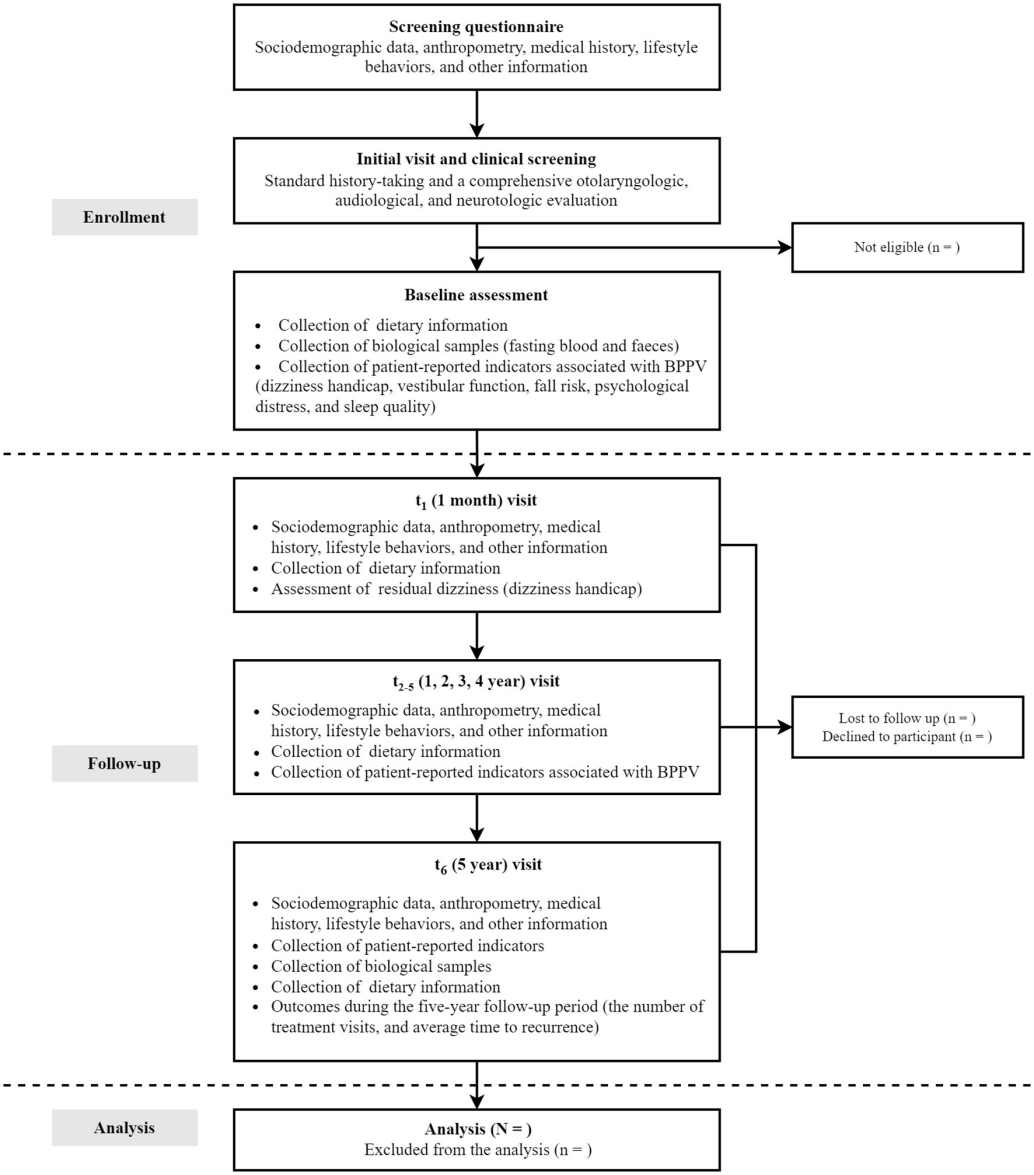
Flowchart of the DaBC design.

Follow-up evaluations occur at 1 month, 1–5 years: 1-month post-baseline through telephone interviews focusing on residual dizziness via DHI updates and dietary adjustments captured by FFQ; Throughout the 1–4 years of follow-up study period, conduct annual post baseline interviews through WeChat or telephone to track changes in lifestyle, recurrent symptoms, FFQ diet, and assess persistent symptoms and related quality of life related to BPPV; and 5 years post-baseline through in-person revisits mirroring baseline procedures, as well as the blood and fecal sample collection. Throughout the 5-year study period, clinical endpoints, including the recurrence and intervals between episodes, are systematically extracted from hospital electronic medical records. Annual reminders via WeChat or phone ensure ongoing participant engagement. The complete study timeline, including enrollment phases, assessment schedules, and data collection protocols, is visually summarized in Figure 3 and operationally detailed in Table 1.

**Table 1:** Data collected in the DaBC cohort study.

**Assessment**	**Study period**						
	**Enrollment**	**Follow-up**					
	*t*_0_	*t*_1_	*t*_2_	*t*_3_	*t*_4_	*t*_5_	*t*_6_
Demographics	√						
Lifestyle behaviors	√	√	√	√	√	√	√
Medical history and medications	√		√	√	√	√	√
Anthropometrics	√	√	√	√	√	√	√
Menopausal status and postmenopausal hormone use (for women)	√	√	√	√	√	√	√
Diet	√	√	√	√	√	√	√
BPPV-related symptoms and signs	√						
Residual dizziness		√	√	√	√	√	√
**BPPV-related characteristics**							
Dizziness Handicap Inventory (DHI)	√	√	√	√	√	√	√
Vestibular Activities of Daily Living Scale (VADL)	√		√	√	√	√	√
Activities-specific Balance Confidence (ABC) Scale	√		√	√	√	√	√
Hospital Anxiety and Depression Scale (HADS)	√		√	√	√	√	√
Pittsburgh Sleep Quality Index (PSQI)	√		√	√	√	√	√
**Biological sample collection and analysis**							
Fasting blood (e.g., neurotransmitters, and biomarkers associated with BPPV)	√						√
DNA extraction and genotyping	√						
Stool samples for microbiome analysis	√						√

t_0_, baseline; t_1_, at 1 month; t_2_, at 1 year; t_3_, at 2 year; t_4_, at 3 year; t_5_, at 4 year; t_6_, at 5 year; BPPV, Benign paroxysmal positional vertigo; DaBC, The Diet and BPPV Cohort Study.

### 2.3 Dietary assessment

**Figure 4:**
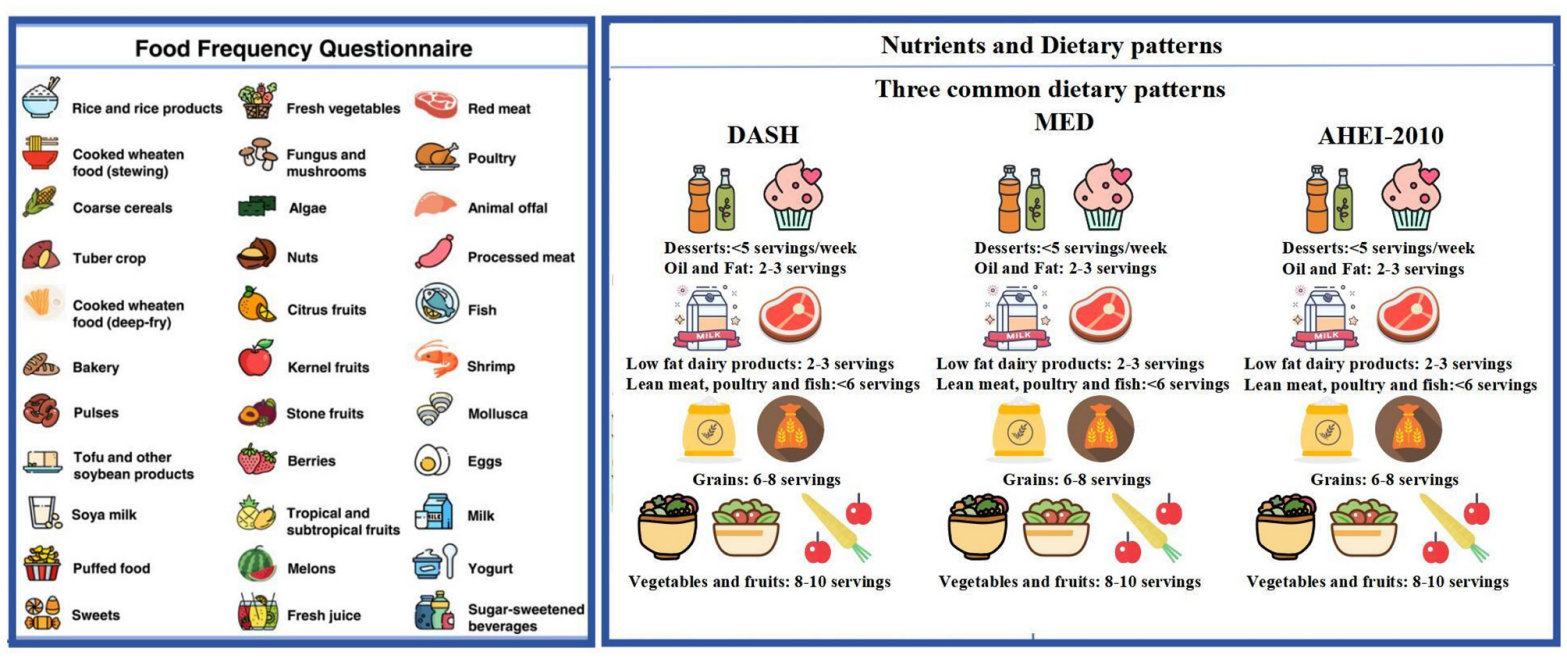
Food frequency questionnaire (FFQ) food, nutrient, and dietary patterns chart.

Dietary patterns promoted in the Dietary Guidelines that are intimately related to disease or crucial biological pathways will be summarized to evaluate the overall quality of the diet. Dietary information is obtained through the FFQ scale at baseline and each follow-up interval. A semi-quantitative FFQ (30) is used to collect detailed dietary intake on the usual consumption frequency of intake of standard portion sizes of each food item during the preceding year. The FFQ is a structured questionnaire on 108 specified food items assembled into 10 categories: cereals; vegetables; fruits; legumes and soybean products; mushrooms and seaweeds; nuts; red meat and processed meat; milk and dairy products; beverages; sweets and baked goods. Participants are required to report the frequency of each food item consumed, selecting from nine predefined frequency categories as follows: “never or less than one serving per month”; “1–3 servings per month”; “1 serving per week”; “2–4 servings per week”; “5–6 servings per week”; “1 serving per day”; “2–3 servings per day”; “4–6 servings per day”; and “6 or more servings per day”. For seasonal foods, participants also indicate the number of months these foods were consumed. The midpoints of the frequency options are utilized to convert consumption frequencies into daily intake frequency, from which the average daily intake of each food in grams (g/day) could be calculated. Furthermore, consumptions of cooking oil and condiments are also investigated. Total daily intakes of energy and nutrients are achieved by multiplying the intake of each food by the portions of nutrients per 100 grams and summing all foods consumed. The structure and content of the FFQ questionnaire are presented in Figure 4.

Dietary patterns promoted in the Dietary Guidelines that are intimately related to disease or crucial biological pathways will be summarized to evaluate the overall quality of the diet. Supplementary Material 1 detailing food mapping decisions, substitutions” and cutoffs for score computation in this cohort is provided.

#### 2.3.1 AHEI-2010

AHEI-2010 evaluated 11 dietary components, emphasizing increased intake of vegetables, fruits, whole grains, nuts, and omega-3 fatty acids. AHEI-2010 emphasized the intake of whole grains, not all grains, and that refined grains do not reduce the risk of metabolic disease, but may increase it. AHEI-2010 recommended, based on the varying health effects of different sources of proteins, that Consumption of nuts, legumes, and fish, especially those rich in EPA + DHA, may reduce the risk of metabolic disease. Meanwhile, AHEI-2010 limits intake of red/processed meats, sugar-sweetened beverages, and sodium. AHEI-2010 suggests that red and processed meats are associated with a higher risk of metabolic disease. Sugar-sweetened beverages are positively associated with the risk of CHD and diabetes. The AHEI-2010 components are scored on a scale of 0 to 10, with a maximum total score of 110. The higher the score, the lower the risk of chronic disease (31).

#### 2.3.2 AMED

AMED assessed 9 components: vegetables, legumes, fruits, nuts, whole grains, red and processed meats, fish, alcohol, and the ratio of monounsaturated to saturated fats. AMED excluded potato products from the vegetable group and assigned a score of 1 to alcohol intake between 5 and 15 g/d. Red and processed meat intakes below the median were assigned 1 point, and other intakes were assigned 0 points. Intake of other components above the median for the study population was scored as 1. AMED scores were associated with lower concentrations of biomarkers of inflammation and endothelial dysfunction, reflecting benefits to cardiovascular and metabolic health (32).

#### 2.3.3 DASH

DASH targets 8 food groups (total fat, saturated fat, protein, fiber, cholesterol, calcium, magnesium, and potassium) with a total score ranging from 0 to 9. One point is assigned to each component when the target is met. If an individual's intake falls between the DASH target and the nutrient content of the DASH control diet, the score for that nutrient is 0.5. The DASH eating pattern reduces blood pressure and cardiovascular risk (33).

#### 2.3.4 EAT-Lancet

The EAT-Lancet diet focuses on 14 foods consisting primarily of vegetables, fruits, whole grains, legumes, nuts, and unsaturated oils, includes small to moderate amounts of seafood and poultry, excludes or consumes small amounts of red meat, processed meats, added sugars, refined grains, and starchy vegetables, and promotes plant-based proteins and a reduction in animal products. It is possible to balance human and planetary health, not only by significantly reducing overall mortality, but also by reducing environmental degradation caused by food production on all scales (34).

#### 2.3.5 LCD

Low-carbohydrate diets (LCDs), characterized by lower carbohydrate intake and higher fat and protein consumption, have been linked to reduced risks of cardiovascular disease and mortality. To assess adherence, we calculated the overall LCD score (OLCDs, range 0–30) using a validated method. Participants were stratified by sex and then divided into 11 quantiles based on the percentage of energy from each macronutrient. Carbohydrate intake was scored from 0 (highest) to 10 (lowest), while protein and fat were scored in reverse. The scores were summed, with higher values indicating greater adherence to a low-carbohydrate pattern (35).

#### 2.3.6 LFD

Low-fat diets (LFDs) aim to reduce saturated and trans fats to lower the risk of chronic diseases, particularly cardiovascular disease, by decreasing fat intake and increasing carbohydrate consumption. To assess adherence, an overall LFD score (OLFDs, range 0–30) will be calculated based on macronutrient energy percentages. Participants will be divided into 11 sex-specific groups for fat, protein, and carbohydrate intake. Higher fat and protein intakes receive higher scores (0–10), while carbohydrate is scored in reverse (higher intake = higher score). The scores are summed, with higher totals indicating greater adherence to a low-fat pattern (36).

#### 2.3.7 PDI

The Plant-based Diet Index (PDI) categorizes 18 foods into healthy and unhealthy plant-based choices. Healthy plant-based foods include whole grains, fruits, vegetables, legumes, vegetable oils, teas, and coffees; unhealthy plant-based foods include refined grains, fruit juices, sugar-sweetened beverages, candies, and desserts. Healthy PDIs can improve human health by boosting fiber, antioxidants, and reducing obesity, among other factors, including lowering diabetes risk, cardiovascular risk, and overall mortality. For the PDI, both healthy and less healthy plant food groups will be assigned positive scores based on quintile intake (Q1 = 1 to Q5 = 5), while animal food groups will be received reverse scores (Q1 = 5 to Q5 = 1). The scores for all 18 food groups are summed to calculate the total PDI score (37).

#### 2.3.8 E-DII

The E-DII quantifies inflammatory potential through 45 dietary components, linking pro-inflammatory diets to chronic disease. Higher scores indicate a more pro-inflammatory diet, and lower scores indicate a more anti-inflammatory diet. The E-DII can help people understand how their diet affects their body's inflammation levels, so they can adjust their diets and choose foods that are more favorable to their health. The E-DII of a pro-inflammatory diet has been associated with an increased risk of obesity, cardiovascular disease, and other diseases (38, 39). Baseline blood samples have been collected and stored, with measurement of serum inflammatory biomarkers (IL-6, CRP, and TNF-α) planned for future work to validate the E-DII (40).

Using a reproducible FFQ, the DaBC framework enabled the longitudinal investigation of associations between changes in intake of food, nutrients, and dietary patterns during a 5-year follow-up and BPPV outcomes (30).

### 2.4 Primary and secondary outcomes

The primary outcome is the number of BPPV relapses during the 5-year follow-up period. A relapse is defined as the recurrence of positional vertigo accompanied by characteristic nystagmus, confirmed through diagnostic maneuvers including the Dix–Hallpike test and supine head roll test (29). During each ENT clinic visit, specialized physicians conduct neurological evaluations and assess potential relapses by synthesizing clinical data: the temporal pattern and frequency of dizziness episodes, nystagmus characteristics observed during diagnostic testing, and resolution status of prior episodes. The secondary outcome is the average recurrence interval during the 5-year follow-up period, which will be calculated as the mean interval between consecutive confirmed relapse episodes based on electronically verified timestamps from hospital records and patient-reported symptom diaries (41, 42).

Moreover, exploratory outcomes are defined as the long-term patient-reported outcomes associated with BPPV, including dizziness handicap, vestibular dysfunction, fall risk, anxiety and depression, and sleep quality. Genome-wide genotyping and analysis of the gut microbiome will also be performed to investigate their potential influence on the association between foods or food groups, nutrients, dietary patterns, and the observed changes in outcomes.

#### 2.4.1 Diagnostic tests

At each visit, the Dix–Hallpike maneuver and the Supine head roll test are performed, and the nystagmus, affected ear or ears, and involved semicircular canals are recorded in detail (29).

##### 2.4.1.1 Dix–Hallpike maneuver

The Dix–Hallpike maneuver is the gold standard for the diagnosis of BPPV of vertical semicircular canals (11), involving a set of specified head movements designed to elicit characteristic nystagmus associated with this condition. The procedure begins with the patient seated upright while the examiner, positioned adjacent to the patient. The examiner rotates the patient's head by 45° to either the left or right to align the posterior semicircular canal with the midsagittal plane of the body. With sustained manual support to maintain the head's 45° rotation, the examiner then transitions the patient from an upright to a supine position, concurrently extending the neck slightly to elevate the chin and allow the head to hang marginally beyond the edge of the examining table (11). The examiner closely observes the patient's eyes for signs of nystagmus, typically a combination of torsional and vertical movements, with the upper pole of the eye veering toward the dependent ear. This maneuver is conducted bilaterally to ascertain the affected ear or ears (11).

##### 2.4.1.2 Supine head roll test

The Supine head roll test is employed to diagnose BPPV of the lateral semicircular canals (43, 44). This test begins with the patient in a supine position, head neutrally aligned, and face upward. The examiner swiftly rotates the patient's head by 90° to one side, monitoring for the onset of horizontal nystagmus, which lacks torsional or vertical components. Following the cessation of nystagmus (or if no nystagmus is caused), the head is returned to the initial supine position. The procedure is then replicated in the opposite direction to evaluate the contralateral ear (43).

#### 2.4.2 Assessment of long-term patient-reported outcomes associated with BPPV

##### 2.4.2.1 Dizziness handicap

The dizziness handicap is quantified using the Dizziness Handicap Inventory (DHI), which comprises 25 questions on physical, functional, and emotional aspects. For each item, patients will be given the following three options: “no”, “sometimes”, or “yes”, respectively assigned 0, 2, 4 points. The total DHI score ranges from 0 to 100, with higher scores indicating a more severe handicap. Based on the total DHI score, the severity of self-perceived dizziness is classified as: mild (0–30), moderate (31–60), and severe (61–100) (45). The Chinese DHI has been demonstrated to have robust test–retest reliability and internal consistency (46).

##### 2.4.2.2 Vestibular function

Vestibular function will be assessed by the Vestibular Activities of Daily Living Scale (VADL), with reliability and validity in evaluating vestibular dysfunction (47). It is a self-administered scale of 28 questions on a 10-point qualitative scale, which is divided into three subscales: functional (items 1–12), ambulation (items 13–21), and instrumental subscale (items 22–28). For each question, the 10 levels of vestibular function-related disability and an inapplicable level will be provided, which are defined in words and with a number to reduce possible ambiguities in interpretation. Median scores are calculated for each subscale and for the total score to assess the ability to perform daily activities and functional activities that challenge balance (47) of participants.

##### 2.4.2.3 Fall risk

Fall risk for BPPV is evaluated using the Activities-specific Balance Confidence (ABC) Scale (48), a 16-item questionnaire that is widely available to measure confidence in maintaining balance across various daily activities. Each activity is rated on a scale from 0% (no confidence) to 100% (complete confidence), with the overall score expressed as an average percentage. A lower percentage score indicates diminished balance confidence and an elevated fall risk.

##### 2.4.2.4 Psychological distress

Psychological distress associated with BPPV is assessed by the Hospital Anxiety and Depression Scale (HADS). Developed by Zigmond and Snaith in 1983, HADS is widely used to identify caseness (possible and probable) of anxiety disorders and depression among patients in non-psychiatric hospital clinics (49). The HADS comprises two subscales (anxiety and depression) with seven items each, scored from 0 (not at all) to 3 (most of the time). A combined subscale of eight or above suggests significant clinical psychiatric symptoms (50).

##### 2.4.2.5 Sleep quality

Sleep quality is measured using the Pittsburgh Sleep Quality Index (PSQI) (51), which evaluates sleep quality and disturbances over the past month. The index consists of 19 items combined into seven components, including sleep duration, sleep disturbance, sleep latency, daytime dysfunction due to sleepiness, sleep efficiency, overall sleep quality, and sleep medication. Each component is scored from 0 (no difficulty) to 3 (severe difficulty), with a total score ranging from 0 to 21. The higher PSQI global score indicates worse sleep quality. A global PSQI score >5 is considered poor sleep quality, validated by its high sensitivity and specificity (51, 52).

#### 2.4.3 Collection and measurement plan of blood samples

Fasting venous blood samples will be obtained by certified phlebotomists. Each participant will provide two 5 mL vials of blood: one in an EDTA-containing tube, and another in a vacutainer tube with a clot activator. EDTA samples will be centrifuged (3,500 g, 15 min, 4 °C) within 30 min to isolate plasma/ buffy coat/ RBCs. The second vial will remain at room temperature for 1 h to coagulate before being centrifuged under the same conditions to separate serum. The processed samples will be aliquoted into 1.5 mL portions, labeled, and stored at −80 °C for future analysis. Plasma levels of neurotransmitters and other bioactive substances will be quantified to investigate their association with BPPV prognosis and the potential mechanisms linking diet and BPPV development (Supplementary material 2).

#### 2.4.4 DNA extraction and genome-wide genotyping

.To comprehensively dissect potential genetic associations in a cohort study of diet and BPPV, DNA Extraction and genome-wide Genotyping will be performed. Initially, DNA will be extracted from the buffy coat using commercial Genomic DNA Extraction kits, following the manufacturer's instructions (53, 54). Subsequently, genome-wide genotyping will be performed using the Affymetrix Axiom^®^ Array with automated procedures. These procedures involve crucial steps such as hybridization, ligation, washing, staining, and scanning using the GeneTitan Instrument. The Axiom GT1 algorithm in the Genotyping Console software will then process the images for genotype analysis. To guarantee the reliability of the genotyping results, quality control filters, including a discordant threshold of 0.82 and a call rate of 97%, will be rigorously applied to ensure genotyping accuracy.

#### 2.4.5 Fecal sample collection and 16S rRNA gene sequencing

To assess potential mediating effects of gut microbiota on the associations between dietary patterns and BPPV recurrence, participants received standardized fecal collection kits for at-home stool sampling, with collection timed within 24 h before clinical assessments. The sample must be transferred to a −80 °C freezer within 2 h of collection for preservation. Fecal DNA will be extracted using a Stool DNA Purification Kit following the prescribed protocol. the V3–V4 region of the bacterial 16S RNA gene will be amplified using unique barcoded primers (338 F and 806 R) via polymerase chain reaction (PCR). The resulting amplicons will be sequenced on an Illumina MiSeq sequencer (55). Bioinformatic analysis will be conducted using the Quantitative Insights into Microbial Ecology (QIIME) platform, encompassing quality filtering of sequences, clustering into operational taxonomic units (OTUs) at a 97% similarity threshold, and classification into taxonomic levels from phylum to genus.

### 2.5 Covariates

Information on demographics (age, sex, educational level, occupation, marital status, residential status, and household income), medical history (cardiovascular disease, diabetes mellitus, liver and kidney function abnormalities, endocrine disorders, anxiety, depression, ocular and auditory conditions, history of malignancies, and traumatic events), family history of chronic disease (hypertension, coronary heart disease, diabetes mellitus, and cancer), anthropometric measures (height, weight), lifestyle behaviors (smoking, alcohol consumption, use of nutritional supplements, exposure to chemical toxins, and daily screen time) and menstruation will be captured by comprehensive questionnaires. The Body mass index (BMI; kg/m^2^) will be calculated as weight in kilograms divided by the square of height in meters. Additionally, physical activity will be assessed using the International Physical Activity Questionnaire (IPAQ) (56) and expressed as metabolic equivalent hours per week (MET hours per week). To mitigate confounding by baseline disease severity, the maximal duration of vertigo episodes was incorporated as a severity marker. This parameter was defined as the longest continuous period of positional vertigo in minutes during the 24 h preceding diagnosis. The selection of vertigo duration as a severity marker aligns with consensus recommendations for BPPV phenotyping (29, 31, 57), where prolonged episodes have demonstrated prognostic relevance for treatment resistance and recurrence risk in prior cohort studies (57).

### 2.6 Data management and confidentiality

A rigorous and standardized research protocol has been scrupulously established, featuring strict standard operating procedures and robust quality control mechanisms. ENT clinicians, as well as neurologists, attend the standardization meetings from the outset and are involved in the diagnosis and the assessment of the prognosis of the BPPV throughout the study. During each visit, study personnel reiterate the study details and follow-up procedures to all participants.

The research team has undergone extensive training to ensure strict adherence to protocol and to enhance the efficiency of the research process. Quality control checks are performed weekly by at least two researchers to identify and rectify any errors, outliers, or missing data. In instances of incomplete or improperly filled questionnaires, the researchers promptly contact participants within a week via telephone to complete and correct the submissions.

Baseline, follow-up, and outcome information are securely stored, accessible only to authorized personnel. Moreover, clinical laboratory measurements and bio-banked samples are managed through a dedicated bio-bank system, which meticulously records details of sample usage and measurements.

### 2.7 Statistical analysis

The distributions of continuous variables for normality are assessed by the Kolmogorov-Smirnov normality test. Descriptive analyses present baseline characteristics of participants by sex as means (standard deviations) for normally distributed variables, medians (interquartile ranges) for skewed variables, and frequency (percentage) for categorical variables. Comparisons between groups at baseline are performed using Student's *t*-test or Wilcoxon rank-sum tests for the continuous variables and the chi-square test for the categorical variables. Sample groupings were trichotomized, with AHEI scores of 42 and 46.4 being used as cutoffs (58, 59), as the AHEI provides a comprehensive measure of overall diet quality, enabling a broader assessment of chronic disease risk (31). Similar analyses will be performed using other dietary indices in subsequent analyses.

Further analysis will categorize participants into tertiles based on dietary pattern scores. Demographic and clinical characteristics will be compared across these categories for class variables, while continuous variables will be analyzed using the Wilcoxon rank-sum test or analysis of variance with subsequent 2 × 2 *post-hoc* comparisons. Multivariate ordinal logistic regression will be used to examine the association between exposure variables and outcome variables (the number of recurrences). Odds ratios (ORs) will be estimated for dietary pattern scores considered as a categorical variable. Linear trends will be assessed using the Wald test by incorporating median values of each tertile of dietary pattern scores as continuous variables in the regression model (60). To explore the dose-response relationships between dietary pattern scores with the average recurrence interval, restricted cubic spline regression with four knots will be used, with scores and outcome analyzed as continuous variables and truncated at the 0.5th and 99.5th centiles. Non-linearity will be evaluated by a likelihood ratio test comparing models with only linear terms against models including both linear and cubic spline terms. Changes in the scoring of long-term patient-reported scale will be calculated as the difference between follow-up and baseline values, and analyzed using linear mixed effects models to assess variations over time across dietary pattern score groups (61). Longitudinal associations between changes in dietary pattern scores and BPPV outcomes are analyzed using generalized estimating equations (GEE) with repeated measures. Dietary pattern scores are categorized into tertiles at each follow-up interval (baseline, 1-year, 5-year), and time-updated tertiles are incorporated as time-varying covariates in the models (62).

Subgroup analyses will stratify participants by baseline covariates, including sex, age, smoking status, BMI, affected semicircular canals, and maximal vertigo episode duration during the 24 h pre-diagnosis (categorized into tertiles: < 2 min, 2–5 min, >5 min) (63).

Genotyping data will be used to estimate the proportion of variance displayed by all single-nucleotide polymorphisms (SNPs) after imputation by restricted maximum-likelihood (64). For microbiome analysis, microbial diversity will be assessed using alpha diversity indices (Shannon and Chao1 index) and beta diversity, which will be visualized through principal component and coordinate analysis. Linear mixed effects models will explore the impacts of genetic variations and gut microbiota on the change in level of related life quality, considering potential interactions (65). Additionally, key neurotransmitters and bioactive substances will be analyzed to explore their mediating effects on the relationships between dietary pattern scores and BPPV prognosis. Multi-omics techniques (including glycomics, lipidomics, proteomics, and microbiomics) and machine learning algorithms will be further integrated into the sample and data analysis (66).

All statistical analyses will be performed with SPSS version 22.0 (SPSS Inc., Chicago, Illinois) or R software, version 4.3.0. A two-sided *p*-value of less than 0.05 will be considered statistically significant.

## 3 Baseline characteristics

Until March 2025, the study had successfully enrolled 844 patients, with a mean age of 50.09 ± 12.27 years, and 420 patients (49.8%) held a bachelor's degree or higher. Age, educational level, household income, age at menarche, body mass index (BMI), medication use, smoking history, and alcohol consumption were comparably distributed across AHEI groups (all *P* > 0.05). Occupational distribution showed significant differences between groups (*P* = 0.01): the AHEI-L (low-score) group had higher proportions of workers (33.70%) and farmers (16.31%), while the AHEI-H (high-score) group had significantly more entrepreneurs/managers (9.54%), government officers (14.84%), and teachers (9.54%). Cardiovascular disease prevalence exhibited a gradient difference (*P* < 0.01), with the AHEI-L group showing the highest rate at 51.4%, significantly higher than the AHEI-M (33.7%) and AHEI-H (17.7%) groups (Table 2).

**Table 2:** The baseline characteristics of population by March 2025.

**Characteristics**	**Total cohort (*n =* 844)**	**AHEI-L (*n =* 282)**	**AHEI-M (*n =* 279)**	**AHEI-H (*n =* 283)**	***P*-value**
Age (years)	50.09 ± 17.27	49.63 ± 13.56	51.17 ± 22.79	49.48 ± 13.90	0.44
Male, *n* (%)	219(26.0)	70(24.8)	76(27.2)	73(25.8)	0.81
Educational level (≥college grade), *n* (%)	420 (49.8)	133 (47.2)	147 (52.7)	140 (49.5)	0.42
Household income (≥5,000 CNY/month), *n* (%)	422 (50.0)	131 (46.5)	149 (53.4)	142 (50.2)	0.26
Occupation, *n* (%)					0.01
Workers	231(27.37)	95 (33.70)	76 (27.24)	60 (21.20)	
Entrepreneurs and managers	59(6.99)	15 (5.32)	17 (6.10)	27 (9.54)	
Government officers	99(11.73)	25 (8.87)	32 (11.47)	42 (14.84)	
Housewives and retirees	181(21.44)	58 (20.57)	60 (21.50)	63 (22.26)	
Teachers	64(7.58)	16 (5.67)	21 (7.53)	27 (9.54)	
Farmers	112(13.27)	46 (16.31)	38 (13.62)	28 (9.89)	
Others	98(11.61)	27 (9.57)	35 (12.54)	36 (12.72)	
Age at menarche (years)	13.97 ± 1.87	13.91 ± 1.80	13.80 ± 1.91	14.20 ± 1.87	0.42
Postmenopausal, *n* (%)	308 (49.3)	105 (37.2)	102 (36.6)	101 (35.7)	0.91
Ever menopausal hormone use, *n* (%)	20 (3.2)	8 (2.8)	7 (2.5)	5 (1.8)	0.70
BMI (kg/m^2^)	22.85 ± 2.96	22.85 ± 3.12	23.00 ± 2.95	22.71 ± 2.81	0.41
Type-2 diabetes mellitus, *n* (%)	55(6.5)	25(8.9)	17(6.1)	13(4.6)	0.24
Cardiovascular disease, *n* (%)	289(34.2)	145(51.4)	94(33.7)	50(17.7)	< 0.01
Medicine use, *n* (%)	297(35.2)	102(36.2)	103(36.9)	92(32.5)	0.50
Smoking history, *n* (%)					0.40
Never smoker	729(86.4)	247(87.6)	236(84.6)	246(86.9)	
Current	85(10.1)	26(9.2)	35(12.5)	24(8.5)	
Former	30(3.6)	9(3.2)	8(2.9)	13(4.6)	
Alcohol consumption, *n* (%)					0.55
No drinking	814(96.4)	269(95.4)	270(96.8)	275(97.2)	
Current	26(3.1)	11(3.9)	7(2.5)	8(2.8)	
Former	4(0.5)	2(0.7)	2(0.7)	-	
Physical activity, MET-h/week	46.30 ± 80.12	52.20 ± 5.30	46.80 ± 82.50	38.10 ± 65.40	0.13

AHEI, alternative health eating index; BMI, body mass index; MET, metabolic equivalents of task.

## 4 Discussion

Given the high risk of recurrence and persistent symptoms following treatment of BPPV, the sustained increase in healthcare utilization has admittedly raised clinical preoccupation (13, 67). Previous epidemiological studies have established associations between dietary components and BPPV, with Schultz et al. (19) reporting significant links between diets deficient in carbohydrates or high in polyunsaturated fatty acids and BPPV incidence. This association may be attributed to vascular damage, ischemia, or atherosclerosis induced by disturbances in glucose and lipid metabolism, resulting in the displacement or degenerative changes of otoconia (68–70). However, the association between dietary habits and the prognosis of BPPV remains poorly understood, despite its importance for a condition characterized by long-term progression. Furthermore, key nutrients, particularly vitamin D (71, 72) and trace elements (73, 74), have been found to exert favorable effects on the recurrence of BPPV by regulating calcium homeostasis and metabolism in the inner ear, as well as ameliorating otolith organ function (73, 75). Wu et al. (76) elucidated that residual dizziness after successful treatment of BPPV is significantly associated with low 25-hydroxyvitamin D [25–(OH)D] levels. Nonetheless, unlike vitamin D, which is notoriously implication in the progression of BPPV, the impact of other nutrients on subsequent patients' manifestations still lacks consensus. Relatively little evidence has been generated for the association between nutrient intake and the detailed health status of BPPV patients after treatment, including vestibular function, fall risk, psychological distress, and sleep quality. Notably, owing to complex nutrient interactions, dietary patterns may better capture the integrated effects of various dietary components and more accurately reflect the multidimensional nature of diet. In a cross-sectional study of 4,183 adults aged 40 years and older in South Korea, a negative association was reported between adherence to a Mediterranean diet and chronic dizziness and imbalance (77), which may imply potential benefits of a balanced diet on BPPV. Nevertheless, to our knowledge, whether recommended healthy dietary patterns drive favorable changes in the progression of BPPV remains largely unexplored. To address these major knowledge gaps, this cohort study aims to investigate the association of foods, food groups, nutrients, and dietary patterns with the risk of recurrence and persistence of symptoms in BPPV.

Current evidence highlights the significance of genetic variations and gut microbiota on the substantial interindividual variability in nutrient intestinal uptake, absorption, metabolism, and tissue response (24, 25, 78). Recent studies have indicated that the mechanism underlying the recurrence of BPPV may be intimately linked to the genetic variants in genes (79, 80). More specifically, the downstream targets of hub genes found to be associated with BPPV progression are enriched in metabolism-related biological processes, such as glucose and lipid metabolism (81). Hence, it is plausible that genetic variants may mediate the relationship between diet and BPPV prognosis, although this remains unclear. Alternatively, gut microbiota dysbiosis has recently been implicated in exacerbating systemic inflammatory responses and disrupting the lymphatic homeostasis in the inner ear via the intestinal microbiota—gut—inner ear axis (82, 83), which may facilitate the suboptimal progression of BPPV. Finally, clinical researchers have identified several paramount neurotransmitters and bioactive substances associated with BPPV, including TPOAb (84), γ-aminobutyrate (85), and Otolin-1 (86), whereas the possible impacts of these known substances on the association of dietary factors with the recurrence of BPPV remain to be elucidated. Therefore, the DaBC study will also evaluate whether the contribution of genotype, intestinal bacteria, and reported bioactive substances modulates the associations between diet and the prognostic burden in BPPV patients.

Our cohort possesses several notable strengths. Firstly, the prospective design, the large sample size, and the use of validated questionnaires will enhance the validity of our study. Moreover, detailed measurements and repeated assessments of a comprehensive set of clinical prognostic indicators allow us to form a detailed picture of the progression of BPPV and promote the translation of the findings into actionable clinical guidance. By integrating dietary and prognostic data with biological samples (blood and stool), various advanced analyses may be conducted (Genome-wide Genotyping, Intestinal flora, biochemical analysis, etc.). Thus, this approach provides an exceptional resource for elucidating the underlying physio-pathological mechanisms and incorporating genetic, microbial, and other relevant data into the investigation of the relationship between nutrition and BPPV recurrence and persistent symptoms. Also, the standardized procedures of data and biospecimen collection, along with a structured follow-up schedule and stringent quality control, ensure the accuracy of data and the reliability of our findings.

Several limitations of the current study deserve consideration. First, due to the observational design of the DaBC study, causal inferences regarding the association between dietary factors and BPPV prognosis cannot be drawn from the cohort data. Second, measurement errors in dietary assessment are inevitable due to the self-reported nature of the questionnaire. However, the FFQ used in our cohort is widely used in population studies to date and has been proven with reasonable reproducibility and validity (30). Third, given the resource constraints, the follow-up period is limited to 5 years, which may not allow us to track the long-term prognosis, although we retained participants' contact information for potential extended follow-up. Fourth, despite adjusting for a wide range of covariates, including sociodemographic, lifestyle, and medical history factors, the possibility of residual or unmeasured confounding affecting the observed associations cannot be fully ruled out. Fifth, selection bias may be a concern, as some participants with incomplete data during recruitment were not included in subsequent follow-up evaluations. Besides, our study only includes BPPV patients, with the lack of a healthy control group at the current stage. Finally, the cohort predominantly consists of participants with Chinese ancestry, which may preclude the generalizability of the findings to other populations. Further studies are warranted to replicate these results in diverse populations.

In summary, the DaBC study will determine whether dietary patterns are associated with the recurrent or persistent symptoms of BPPV, while exploring the potential effects that derive from specific modulations of genetic variations and gut microbiota. Findings from this study may serve to shed new light on optimal dietary choices for BPPV patients, so as to improve the long-term prognosis, and contribute to the development of clinical practice guidelines for BPPV management.

## Data Availability

The raw data supporting the conclusions of this article will be made available by the authors, without undue reservation.
