# Supplementary material for: The diet and benign paroxysmal positional vertigo (DaBC) study: protocol and baseline characteristics of a prospective cohort investigating dietary patterns and BPPV prognosis—the role of genetics and gut microbiota
Source: Front Nutr. 2025 Sep 15;12:1640153. doi: 10.3389/fnut.2025.1640153 (PMC12477046; doi:10.3389/fnut.2025.1640153)
Supplement: Supplementary file 1 [file Table_1.docx]

**Supplementary Materials 1**

**Cohort Profile: The DaBC Cohort**

**Journal：**Frontiers in Nutrition

**Authors**:

Juanli Xing^1#^, Hongying Shan^2#^, Xinyu Xu^2,3,4#^, Wenyan Shi^4^, Peihua Ren^2,5^, Jiaqian Wu^4^, Le Ma^2*^, Baibing Mi^2*^

***Correspondence**:

Baibing Mi

Department of Epidemiology and Biostatistics Xi'an Jiaotong University Health Science Center, Shaanxi, Xi'an, China. No. 76, Yanta West Road, Xi’an, Shaanxi, China.Email: [xjtu.mi@xjtu.edu.cn](mailto:xjtu.mi@xjtu.edu.cn)

Le Ma

Department of Epidemiology and Biostatistics Xi'an Jiaotong University Health Science Center, Shaanxi, Xi'an, China. No. 76, Yanta West Road, Xi’an, Shaanxi, China.Email:male@mail.xjtu.edu.cn

**Supplementary Table S1. Dietary components and scoring algorithms**

| **Diet score and components** | **Food items** | **Score range** | **Standard for maximum score** | **Standard for minimum score** |
| --- | --- | --- | --- | --- |
| **AHEI-2010(1)** |  |  |  |  |
| Vegetables | Cabbage/kale, lettuce, spinach, watercress, rapeseed, chrysanthemum greens, celery, carrots, butternut squash, turnip, chinese chives, other vegetables | 0-10 | 0 | ≥5 |
| Fruit | Pear, apple, peach/nectarine, plum, orange, orange-like small fruits,mango, banana, melon, pomelo, pineapple, grape, cherry, strawberry, pitaya, kiwi, other fruits | 0-10 | 0 | ≥4 |
| Whole grains,g/d | Wheat, corn, other cooked grains |  |  |  |
| Women |  | 0-10 | 0 | 75 |
| Men |  | 0-10 | 0 | 90 |
| Sugar-sweetened beverages and fruit juice | Orange juice, fizzy drink, fruit smoothie, dairy smoothie,other sugar-sweetened beverages | 0-10 | ≥1 | 0 |
| Nuts and legumes | Salted peanuts, unsalted peanuts, walnut, seeds, beans (baked beans), other beans or lentils, tofu, soy milk, other soy products | 0-10 | 0 | ≥1 |
| Red/processed meat | Beef, pork, lamb, sausage, bacon, ham | 0-10 | ≥1.5 | 0 |
| trans Fat (% of energy) | — | 0-10 | ≥4 | ≤0.5 |
| Long-chain (n-3) fats (EPA + DHA) (mg/d) | — | 0-10 | 0 | 250 |
| PUFA (% of energy)energy | — | 0-10 | ≤2 | ≥10 |
| Sodium (mg/d) | — | 0-10 | 19.27 | 4.34 |
| Alcohol, drinks/d ^*^ | Beer, red wine, white wine |  |  |  |
| Women |  | 0-10 | ≥2.5 | 0.5–1.5 |
| Men |  | 0-10 | ≥3.5 | 0.5–2.0 |
| **AMED(2)** |  |  | (median) | (median) |
| Whole grains | Wheat, corn, other cooked grains | 0 or 1 | ≥0.05 | < 0.05 |
| Fruits | Pear, apple, peach/nectarine, plum, orange, orange-like small fruits,mango, banana, melon, pomelo, pineapple, grape, cherry, strawberry, pitaya, kiwi, other fruits | 0 or 1 | ≥0.05 | < 0.05 |
| Vegetables | Cabbage/kale, potato, lettuce, spinach, watercress, chinese chives, rapeseed, chrysanthemum greens, celery, carrots, sweet potato, butternut squash, turnip, other vegetables | 0 or 1 | ≥0.79 | < 0.79 |
| Nuts | Salted peanuts, unsalted peanuts, walnut, seeds | 0 or 1 | ≥0.05 | < 0.05 |
| Legumes | Beans (baked beans), other beans or lentils, tofu, soy milk, other soy products | 0 or 1 | ≥0.05 | < 0.05 |
| Fish | Tinned tuna, oily fish, breaded fish, battered fish, white fish, carp, catfish, trout, salmon, cod, prawns, lobster/crab, shellfish, other fish intake | 0 or 1 | ≥0.05 | < 0.05 |
| Read meat | Beef, pork, lamb | 0 or 1 | ≥0.42 | < 0.42 |
| Alcohol, g/day |  | 0 or 1 | <5 or>15 | 5-15 |
| Ratio of MUFA to SFA | — | 0 or 1 | < 1.10 | ≥1.10 |
| **DASH** |  |  |  |  |
| Saturated fat (% of energy) | — | 0-1 | ≤6 | >11% |
| Total fat (% of energy) | — | 0-1 | ≤27 | >32% |
| Protein (% of energy) | — | 0-1 | ≥18 | <16.5 |
| Cholesterol (mg/1000 kcal) | — | 0-1 | ≤71.4 | >107.1 |
| Fiber (g/1000 kcal) | — | 0-1 | ≥14.8 | <9.5 |
| Magnesium (mg/1000 kcal) | — | 0-1 | ≥238 | <158 |
| Calcium (mg/1000 kcal) | — | 0-1 | ≥590 | <402 |
| Potassium (mg/1000 kcal) | — | 0-1 | ≥2238 | <1534 |
| Sodium (mg/1000 kcal) | — | 0-1 | ≤2400 | >2800 |
| **EAT-Lancet** |  |  |  |  |
| Whole grains (g/day) | Wheat, corn, other cooked grains | 0-10 | 0 | 232 |
| Tubers (g/day) | Potato, sweet potato, other tubers | 0-10 | ≥200 | ≤50 |
| All vegetables (g/day) | Cabbage/kale, potato, lettuce, spinach, watercress, chinese chives, rapeseed, chrysanthemum greens, celery, carrots, sweet potato, butternut squash, turnip, other vegetables | 0-10 | 0 | ≥300 |
| Fruits (g/day) | Pear, apple, peach/nectarine, plum, orange, orange-like small fruits,mango, banana, melon, pomelo, pineapple, grape, cherry, strawberry, pitaya, kiwi, other fruits | 0-10 | 0 | ≥200 |
| Dairy foods (g/day) | Milk, milk powder, yogurt | 0-10 | ≥1000 | ≤250 |
| Red or processed meat (g/day) | Beef, pork, lamb, sausage, bacon, ham | 0-10 | ≥100 | ≤14 |
| Poultry (g/day) | Chicken, duck and goose meat | 0-10 | ≥100 | ≤29 |
| Eggs (g/day) | Whole eggs | 0-10 | ≥120 | ≤13 |
| Fish (g/day) | Tinned tuna, oily fish, breaded fish, battered fish, white fish, carp, catfish, trout, salmon, cod, prawns, lobster/crab, shellfish, other fish intake | 0-10 | 0 | ≥28 |
| Nuts (g/day) | Salted peanuts, unsalted peanuts, walnut, seeds | 0-10 | 0 | ≥50 |
| Legumes and soy foods (g/day) | Beans (baked beans), other beans or lentils, tofu, soy milk, other soy products | 0-10 | 0 | ≥150 |
| Saturated fats (% of energy) | — | 0-10 | ≥10 | 0 |
| Unsaturated fats (% of energy) | — | 0-10 | ≤3.5 | ≥21 |
| All sweeteners (% of energy) | Cakes, cookies, candies, chocolates,preserved fruits, other sweets | 0-10 | ≥25 | ≤5 |
| **LCD** |  |  |  |  |
| Carbohydrate (%) | — | 0-10 | <40.59 (Lowest undecile) | >56.8 (Highest undecile) |
| Protein (%) | — | 0-10 | >13.89 (Highest undecile) | <8.36 (Lowest undecile) |
| Fat (%) | — | 0-10 | >38.53 (Highest undecile) | <23.7 (Lowest undecile) |
| **LFD** |  |  |  |  |
| Carbohydrate (%) | — | 0-10 | >56.8 (Highest undecile) | <40.59 (Lowest undecile) |
| Protein (%) | — | 0-10 | >13.89 (Highest undecile) | <8.36 (Lowest undecile) |
| Fat (%) | — | 0-10 | <23.7 (Lowest undecile) | >38.53 (Highest undecile) |
| **PDI** |  |  |  |  |
| Healthy Plant Food Groups |  |  | (Highest quintile) | (Lowest quintile) |
| Whole grains | Wheat, corn, other cooked grains | 0-10 | >0.43 | 0 |
| Fruits | Pear, apple, peach/nectarine, plum, orange, orange-like small fruits,mango, banana, melon, pomelo, pineapple, grape, cherry, strawberry, pitaya, kiwi, other fruits | 0-10 | >0.14 | 0 |
| Vegetables | Cabbage/kale, lettuce, spinach, watercress, chinese chives, rapeseed, chrysanthemum greens, celery, carrots, sweet potato, butternut squash, turnip, other vegetables | 0-10 | >1 | <0.14 |
| Nuts | Salted peanuts, unsalted peanuts, walnut, seeds | 0-10 | >0.14 | 0 |
| Legumes | Beans (baked beans), other beans or lentils, tofu, soy milk, other soy products | 0-10 | >0.14 | 0 |
| Vegetable oils (g/month) | Vegetable oils | 0-10 | >2000 | <100 |
| Tea & Coffee | Standard tea, rooibos tea, green tea, herbal tea, other tea, instant coffee, filtered coffee, cappuccino, latte, espresso, other coffee drinks | 0-10 | >0.05 | 0 |
| Less Healthy Plant Food Groups |  |  | (Highest quintile) | (Lowest quintile) |
| Fruit juices | Orange juice | 0-10 | >0.05 | 0 |
| Refined grains | Rice, stuffed buns, dumplings, white bread, sliced bread, chinese pastries | 0-10 | >1 | <0.14 |
| Potatoes | Potatoes | 0-10 | >0.43 | <0.05 |
| Sugar-sweetened beverages | Fizzy drink, fruit smoothie, dairy smoothie,other sugar-sweetened beverages | 0-10 | >0.05 | 0 |
| Sweets and Desserts | Cakes, cookies, candies, chocolates,preserved fruits, other sweets | 0-10 | >0.05 | 0 |
| Animal Food Groups |  |  | (Lowest quintile) | (Highest quintile) |
| Animal fat (g/month) | Animal oil | 0-10 | 0 | >100 |
| Dairy | Milk, milk powder, yogurt | 0-10 | 0 | >0.43 |
| Egg | Whole eggs | 0-10 | <0.14 | >1 |
| Fish or Seafood | Tinned tuna, oily fish, breaded fish, battered fish, white fish, carp, catfish, trout, salmon, cod, prawns, lobster/crab, shellfish, other fish intake | 0-10 | 0 | >0.05 |
| Meat | Beef, pork, lamb, poultry, sausage, bacon, ham, animal offal | 0-10 | 0 | >0.43 |
| Misc. animal-based foods | Pizza | 0-10 | 0 | ≥0 |
| **E-DII** |  |  |  |  |
| Processed meant | Sausage, bacon, ham | 0-1.38 | ≥1 | 0 |
| Red meat | Beef, pork, lamb |  |  |  |
| Women |  | 0-1.33 | ≥0.5 | 0 |
| Men |  | 0-1.33 | ≥0.75 | 0 |
| Organ meat | Animal offal | 0 - 0.4 | >0 | 0 |
| Fish | Tinned tuna, oily fish, breaded fish, battered fish, white fish, carp, catfish, trout, salmon, cod, prawns, lobster/crab, shellfish, other fish intake | 0- 0.35 | ≥0.5 | 0 |
| Dark yellow vegetables | Carrots, sweet potato, butternut squash, turnip |  |  |  |
| Women |  | -0.78 - 0 | 0 | >0.75 |
| Men |  | -0.78 - 0 | 0 | ≥0.5 |
| Green leafy vegetables | Cabbage/kale, lettuce, spinach, watercress, chinese chives, rapeseed, chrysanthemum greens, celery |  |  |  |
| Women |  | -0.78 -0 | 0 | >0.6 |
| Men |  | -0.78 -0 | 0 | ≥0.5 |
| Other vegetables | Other vegetables |  |  |  |
| Women |  | 0-0.4 | ≥3.5 | 0 |
| Men |  | 0-0.4 | ≥3.1 | 0 |
| Refined grains | Rice, stuffed buns, dumplings, white bread, sliced bread, chinese pastries |  |  |  |
| Women |  | 0-1.62 | ≥4 | 0 |
| Men |  | 0-1.62 | ≥5 | 0 |
| High-energy beverages | Fizzy drink, fruit smoothie, dairy smoothie,other high-energy beverages |  |  |  |
| Women |  | 0-1.5 | ≥0.25 | 0 |
| Men |  | 0-1.5 | ≥0.33 | 0 |
| Low-energy beverages | Low-energy beverages |  |  |  |
| Women |  | 0-1.1 | ≥0.25 | 0 |
| Men |  | 0-1.1 | >0 | 0 |
| Tomato | Tomato |  |  |  |
| Women |  | 0-0.92 | ≥1 | 0 |
| Men |  | 0-0.92 | ≥0.56 | 0 |
| Beer | Beer |  |  |  |
| Women |  | -0.16-0 | 0 | >0 |
| Men |  | -0.16-0 | 0 | ≥1 |
| Wine | Red wine, white wine |  |  |  |
| Women |  | -2.11-0 | 0 | >1.5 |
| Men |  | -2.11-0 | 0 | ≥2 |
| Tea | Standard tea, rooibos tea, green tea, herbal tea, other tea |  |  |  |
| Women |  | 0-0.11 | ≥4.5 | 0 |
| Men |  | 0-0.11 | ≥4 | 0 |
| Coffee | Instant coffee, filtered coffee, cappuccino, latte, espresso,  other coffee drinks | -2.83-0 | 0 | ≥3 |
| Fruit juices | Orange juice | -0.22-0 | 0 | ≥1 |
| Snacks | Cakes, cookies, candies, chocolates,preserved fruits, other sweets | -0.33-0 | 0 | ≥0.5 |
| Pizza | Pizza | -0.44-0 | 0 | ≥0 |

All diet scores are calculated by adding the number of points awarded proportionally across the indicated ranges. Standard for maximum score and for minimum score are in servings/ day unless otherwise indicated. Specified servings sizes are indicated in parentheses. Quintiles (fifths) of intake. Undeciles: eleven groups with equal sample sizes. *: One drink is 4 oz of wine, 12 oz of beer, or 1.5 oz of liquor (1 oz = 28.35 g).
