# Supplementary material for: The diet and benign paroxysmal positional vertigo (DaBC) study: protocol and baseline characteristics of a prospective cohort investigating dietary patterns and BPPV prognosis—the role of genetics and gut microbiota
Source: Front Nutr. 2025 Sep 15;12:1640153. doi: 10.3389/fnut.2025.1640153 (PMC12477046; doi:10.3389/fnut.2025.1640153)
Supplement: Supplementary file 2 [file Table_2.pdf]

Supplementary Table S2. Dietary components and scoring algorithms

Diet score and components	Food items	Score range	Standard for maximum score	Standard for minimum score
**AHEI-2010(1)**				
Vegetables	Cabbage/kale, lettuce, spinach, watercress, rapeseed, chrysanthemum greens, celery, carrots, butternut squash, turnip, chinese chives, other vegetables	0-10	0	≥5
Fruit	Pear, apple, peach/nectarine, plum, orange, orange-like small fruits,mango, banana, melon, pomelo, pineapple, grape, cherry, strawberry, pitaya, kiwi, other fruits	0-10	0	≥4
Whole grains,g/d	Wheat, corn, other cooked grains			
Women		0-10	0	75
Men		0-10	0	90
Sugar-sweetened beverages and fruit juice	Orange juice, fizzy drink, fruit smoothie, dairy smoothie,other sugar-sweetened beverages	0-10	≥1	0
Nuts and legumes	Salted peanuts, unsalted peanuts, walnut, seeds, beans (baked beans), other beans or lentils, tofu, soy milk, other soy products	0-10	0	≥1
Red/processed meat	Beef, pork, lamb, sausage, bacon, ham	0-10	≥1.5	0
trans Fat (% of energy)	—	0-10	≥4	≤0.5
Long-chain (n-3) fats (EPA + DHA) (mg/d)	—	0-10	0	250
PUFA (% of energy)energy	—	0-10	≤2	≥10
Sodium (mg/d)	—	0-10	19.27	4.34

Alcohol, drinks/d *	Beer, red wine, white wine			
Women		0-10	≥2.5	0.5–1.5
Men		0-10	≥3.5	0.5–2.0
**AMED(2)**			(median)	(median)
Whole grains	Wheat, corn, other cooked grains	0 or 1	≥0.05	< 0.05
Fruits	Pear, apple, peach/nectarine, plum, orange, orange-like small fruits,mango, banana, melon, pomelo, pineapple, grape, cherry, strawberry, pitaya, kiwi, other fruits	0 or 1	≥0.05	< 0.05
Vegetables	Cabbage/kale, potato, lettuce, spinach, watercress, chinese chives, rapeseed, chrysanthemum greens, celery, carrots, sweet potato, butternut squash, turnip, other vegetables	0 or 1	≥0.79	< 0.79
Nuts	Salted peanuts, unsalted peanuts, walnut, seeds	0 or 1	≥0.05	< 0.05
Legumes	Beans (baked beans), other beans or lentils, tofu, soy milk, other soy products	0 or 1	≥0.05	< 0.05
Fish	Tinned tuna, oily fish, breaded fish, battered fish, white fish, carp, catfish, trout, salmon, cod, prawns, lobster/crab, shellfish, other fish intake	0 or 1	≥0.05	< 0.05
Read meat	Beef, pork, lamb	0 or 1	≥0.42	< 0.42
Alcohol, g/day		0 or 1	<5 or>15	5-15
Ratio of MUFA to SFA	—	0 or 1	< 1.10	≥1.10
**DASH**				
Saturated fat (% of energy)	—	0-1	≤6	>11%
Total fat (% of energy)	—	0-1	≤27	>32%
Protein (% of energy)	—	0-1	≥18	<16.5

Cholesterol (mg/1000 kcal)	—	0-1	≤71.4	>107.1
Fiber (g/1000 kcal)	—	0-1	≥14.8	<9.5
Magnesium (mg/1000 kcal)	—	0-1	≥238	<158
Calcium (mg/1000 kcal)	—	0-1	≥590	<402
Potassium (mg/1000 kcal)	—	0-1	≥2238	<1534
Sodium (mg/1000 kcal)	—	0-1	≤2400	>2800
**EAT-Lancet**				
Whole grains (g/day)	Wheat, corn, other cooked grains	0-10	0	232
Tubers (g/day)	Potato, sweet potato, other tubers	0-10	≥200	≤50
All vegetables (g/day)	Cabbage/kale, potato, lettuce, spinach, watercress, chinese chives, rapeseed, chrysanthemum greens, celery, carrots, sweet potato, butternut squash, turnip, other vegetables	0-10	0	≥300
Fruits (g/day)	Pear, apple, peach/nectarine, plum, orange, orange-like small fruits,mango, banana, melon, pomelo, pineapple, grape, cherry, strawberry, pitaya, kiwi, other fruits	0-10	0	≥200
Dairy foods (g/day)	Milk, milk powder, yogurt	0-10	≥1000	≤250
Red or processed meat (g/day)	Beef, pork, lamb, sausage, bacon, ham	0-10	≥100	≤14
Poultry (g/day)	Chicken, duck and goose meat	0-10	≥100	≤29
Eggs (g/day)	Whole eggs	0-10	≥120	≤13
Fish (g/day)	Tinned tuna, oily fish, breaded fish, battered fish, white fish, carp, catfish, trout, salmon, cod, prawns, lobster/crab, shellfish, other fish intake	0-10	0	≥28
Nuts (g/day)	Salted peanuts, unsalted peanuts, walnut, seeds	0-10	0	≥50
Legumes and soy foods (g/day)	Beans (baked beans), other beans or lentils, tofu, soy	0-10	0	≥150

	milk, other soy products			
Saturated fats (% of energy)	—	0-10	≥10	0
Unsaturated fats (% of energy)	—	0-10	≤3.5	≥21
All sweeteners (% of energy)	Cakes, cookies, candies, chocolates,preserved fruits, other sweets	0-10	≥25	≤5
**LCD**				
Carbohydrate (%)	—	0-10	<40.59 (Lowest undecile)	>56.8 (Highest undecile)
Protein (%)	—	0-10	>13.89 (Highest undecile)	<8.36 (Lowest undecile)
Fat (%)	—	0-10	>38.53 (Highest undecile)	<23.7 (Lowest undecile)
**LFD**				
Carbohydrate (%)	—	0-10	>56.8 (Highest undecile)	<40.59 (Lowest undecile)
Protein (%)	—	0-10	>13.89 (Highest undecile)	<8.36 (Lowest undecile)
Fat (%)	—	0-10	<23.7 (Lowest undecile)	>38.53 (Highest undecile)
**PDI**				
Healthy Plant Food Groups			(Highest quintile)	(Lowest quintile)
Whole grains	Wheat, corn, other cooked grains	0-10	>0.43	0
Fruits	Pear, apple, peach/nectarine, plum, orange, orange-like small fruits,mango, banana, melon, pomelo, pineapple, grape, cherry, strawberry, pitaya, kiwi, other fruits	0-10	>0.43	0
Vegetables	Cabbage/kale, lettuce, spinach, watercress, chinese chives, rapeseed, chrysanthemum greens, celery, carrots, sweet potato, butternut squash, turnip, other vegetables	0-10	>1	<0.14
Nuts	Salted peanuts, unsalted peanuts, walnut, seeds	0-10	>0.14	0
Legumes	Beans (baked beans), other beans or lentils, tofu, soy	0-10	>0.14	0

	milk, other soy products			
Vegetable oils (g/month)	Vegetable oils	0-10	>2000	<60
Tea & Coffee	Standard tea, rooibos tea, green tea, herbal tea, other tea, instant coffee, filtered coffee, cappuccino, latte, espresso, other coffee drinks	0-10	>0.05	0
Less Healthy Plant Food Groups			(Highest quintile)	(Lowest quintile)
Fruit juices	Orange juice	0-10	>0.05	0
Refined grains	Rice, stuffed buns, dumplings, white bread, sliced bread, chinese pastries	0-10	>1	<0.14
Potatoes	Potatoes	0-10	>0.43	<0.05
Sugar-sweetened beverages	Fizzy drink, fruit smoothie, dairy smoothie,other sugar-sweetened beverages	0-10	>0.05	0
Sweets and Desserts	Cakes, cookies, candies, chocolates,preserved fruits, other sweets	0-10	>0.05	0
Animal Food Groups			(Lowest quintile)	(Highest quintile)
Animal fat (g/month)	Animal oil	0-10	0	>100
Dairy	Milk, milk powder, yogurt	0-10	0	>0.43
Egg	Whole eggs	0-10	<0.14	>1
Fish or Seafood	Tinned tuna, oily fish, breaded fish, battered fish, white fish, carp, catfish, trout, salmon, cod, prawns, lobster/crab, shellfish, other fish intake	0-10	0	>0.05
Meat	Beef, pork, lamb, poultry, sausage, bacon, ham, animal offal	0-10	0	>0.43
Misc. animal-based foods	Pizza	0-10	0	≥0

E-DII

Processed meant	Sausage, bacon, ham	0-1.38	≥1	0
Red meat	Beef, pork, lamb			
Women		0-1.33	≥0.5	0
Men		0-1.33	≥0.75	0
Organ meat	Animal offal	0 - 0.4	>0	0
Fish	Tinned tuna, oily fish, breaded fish, battered fish, white fish, carp, catfish, trout, salmon, cod, prawns, lobster/crab, shellfish, other fish intake	0- 0.35	≥0.5	0
Dark yellow vegetables	Carrots, sweet potato, butternut squash, turnip			
Women		-0.78 - 0	0	>0.75
Men		-0.78 - 0	0	≥0.5
Green leafy vegetables	Cabbage/kale, lettuce, spinach, watercress, chinese chives, rapeseed, chrysanthemum greens, celery			
Women		-0.78 -0	0	>0.6
Men		-0.78 -0	0	≥0.5
Other vegetables	Other vegetables			
Women		0-0.4	≥3.5	0
Men		0-0.4	≥3.1	0
Refined grains	Rice, stuffed buns, dumplings, white bread, sliced bread, chinese pastries			
Women		0-1.62	≥4	0
Men		0-1.62	≥5	0
High-energy beverages	Fizzy drink, fruit smoothie, dairy smoothie,other high-energy beverages			
Women		0-1.5	≥0.25	0

Men		0-1.5	≥0.33	0
Low-energy beverages	Low-energy beverages			
Women		0-1.1	≥0.25	0
Men		0-1.1	>0	0
Tomato	Tomato			
Women		0-0.92	≥1	0
Men		0-0.92	≥0.56	0
Beer	Beer			
Women		-0.16-0	0	>0
Men		-0.16-0	0	≥1
Wine	Red wine, white wine			
Women		-2.11-0	0	>1.5
Men		-2.11-0	0	≥2
Tea	Standard tea, rooibos tea, green tea, herbal tea, other tea			
Women		0-0.11	≥4.5	0
Men		0-0.11	≥4	0
Coffee	Instant coffee, filtered coffee, cappuccino, latte, espresso, other coffee drinks	-2.83-0	0	≥3
Fruit juices	Orange juice	-0.22-0	0	≥1
Snacks	Cakes, cookies, candies, chocolates,preserved fruits, other sweets	-0.33-0	0	≥0.5
Pizza	Pizza	-0.44-0	0	≥0

All diet scores are calculated by adding the number of points awarded proportionally across the indicated ranges. Standard for maximum score and for minimum score are in servings/ day unless otherwise indicated. Specified servings sizes are indicated in parentheses. Quintiles (fifths) of intake. Undeciles: eleven groups with

equal sample sizes. *: One drink is 4 oz of wine, 12 oz of beer, or 1.5 oz of liquor (1 oz = 28.35 g).
